# A Standardized Needs Assessment Tool to Inform the Curriculum Development Process for Pediatric Resuscitation Simulation-Based Education in Resource-Limited Settings

**DOI:** 10.3389/fped.2018.00037

**Published:** 2018-02-28

**Authors:** Nicole Shilkofski, Amanda Crichlow, Julie Rice, Leslie Cope, Ye Myint Kyaw, Thazin Mon, Sarah Kiguli, Julianna Jung

**Affiliations:** ^1^Department of Pediatrics, Johns Hopkins University School of Medicine, Baltimore, MD, United States; ^2^Department of Emergency Medicine, University of Florida College of Medicine, Jacksonville, FL, United States; ^3^Department of Emergency Medicine, Johns Hopkins University School of Medicine, Baltimore, MD, United States; ^4^Department of Oncology, Johns Hopkins University School of Medicine, Division of Bioinformatics and Biostatistics, Baltimore, MD, United States; ^5^Head of Department of Pediatrics, University of Medicine 1 Yangon, Yangon Children’s Hospital, Yangon, Myanmar; ^6^Department of Pediatrics, University of Medicine 2 Yangon, Yangon Children’s Hospital, Yangon, Myanmar; ^7^Department of Pediatrics, Makerere University College of Health Sciences, Kampala, Uganda

**Keywords:** pediatric resuscitation, simulation-based training, limited-resource settings, pediatric critical care, needs assessment, neonatal resuscitation, developing countries, PIPES tool

## Abstract

**Introduction:**

Under five mortality rates (UFMR) remain high for children in low- and middle-income countries (LMICs) in the developing world. Education for practitioners in these environments is a key factor to improve outcomes that will address United Nations Sustainable Development Goals 3 and 10 (good health and well being and reduced inequalities). In order to appropriately contextualize a curriculum using simulation, it is necessary to first conduct a needs assessment of the target learner population. The World Health Organization (WHO) has published a tool to assess capacity for emergency and surgical care in LMICs that is adaptable to this goal.

**Materials and methods:**

The WHO Tool for Situational Analysis to Assess Emergency and Essential Surgical Care was modified to assess pediatric resuscitation capacity in clinical settings in two LMICs: Uganda and Myanmar. Modifications included assessment of self-identified learning needs, current practices, and perceived epidemiology of disease burden in each clinical setting, in addition to assessment of pediatric resuscitation capacity in regard to infrastructure, procedures, equipment, and supplies. The modified tool was administered to 94 respondents from the two settings who were target learners of a proposed simulation-based curriculum in pediatric and neonatal resuscitation.

**Results:**

Infectious diseases (respiratory illnesses and diarrheal disease) were cited as the most common causes of pediatric deaths in both countries. Self-identified learning needs included knowledge and skill development in pediatric airway/breathing topics, as well as general resuscitation topics such as CPR and fluid resuscitation in shock. Equipment and supply availability varied substantially between settings, and critical shortages were identified in each setting. Current practices and procedures were often limited by equipment availability or infrastructural considerations.

**Discussion and conclusion:**

Epidemiology of disease burden reported by respondents was relatively consistent with WHO country-specific UFMR statistics in each setting. Results of the needs assessment survey were subsequently used to refine goals and objectives for the simulation curriculum and to ensure delivery of pragmatic educational content with recommendations that were contextualized for local capacity and resource availability. Effective use of the tool in two different settings increases its potential generalizability.

## Introduction

Globally, 5.6 million children under the age of 5 years died in 2016 (1). Since 1990, early childhood mortality has decreased substantially around the world, from 98 to 41 of every 1,000 live births. However, this rate remains much higher than the United Nations Sustainable Development Goal target of 25/1,000, and significant progress is needed to achieve this objective (2, 3). For the past two decades, it has been recognized that a multitude of factors contribute to potentially preventable child deaths in low- and middle-income countries (LMICs) (4). One thing that has not changed among these factors is the lack of high quality and contextually appropriate educational curricula to teach provision of care to critically ill children in these settings.

The World Health Organization (WHO) has developed the integrated management of childhood illness (IMCI) strategy and the emergency triage assessment and treatment (ETAT) guidelines for use in resource-limited settings (5, 6). Despite the demonstrated feasibility and success of some of these initiatives in LMICs (7), adherence to these guidelines by medical providers is highly variable (8, 9). Provider education has been shown to improve quality of care, but even among trained providers, performance remains suboptimal, suggesting the need for additional educational reinforcement (10).

For this reason, effective resuscitation education programs are needed in LMICs. Some training programs have been designed specifically for use in resource-limited settings, such as the Helping Babies Breathe (HBB) and Helping Mothers Survive (HMS) Bleeding after Birth initiatives (11–16). However, other resuscitation courses, such as the American Heart Association (AHA) Pediatric Advanced Life Support (PALS) and Pediatric Emergency Assessment Recognition and Stabilization (PEARS), have not been fully adapted for use in resource-limited settings. This has led many investigators to undertake these adaptations on an individual basis, often without a clear process to guide their efforts (17). However, “contextualized training,” defined as “local adaptation of training, utilizing existing and sustainable resources for both care and training,” has been associated with increases in provider confidence and short-term knowledge retention (18).

Contextualization of training requires rigorous understanding of the target learners and local clinical environment. In the curriculum development process, this understanding is gained through a needs assessment, which allows educators to ensure that curricula are appropriate and relevant to the target learner group (19). Needs assessment is particularly critical in international education, where epidemiology, resource availability, and practice standards vary widely (20). While several needs assessment surveys addressing critical care capacity have been described in the literature (21–25), there is presently no validated and comprehensive tool that is specific to pediatric resuscitation education in LMICs.

The WHO developed a tool in 2008 to measure surgical capacity (the Tool for Situational Analysis to Assess Emergency and Essential Surgical Care) (26), which has been validated and used in more than 10 LMICs (27–37). It was subsequently modified to develop a survey for specific facilities based on personnel, infrastructure, procedures, equipment, and supplies (PIPES) (38). PIPES has been used with further modifications to assess pediatric surgical capacity in 37 hospitals in West Africa (PediPIPES) (39).

In order to garner information needed to develop an appropriately contextualized pediatric resuscitation curriculum for use in two LMIC settings, we adapted the PIPES and PediPIPES to construct a novel needs assessment tool. Our immediate goal in developing this new tool was to evaluate local pediatric emergency care capacity, resources, and practices (PIPES), as well as perceived causes of pediatric morbidity and mortality, and self-assessed educational needs of target learners. Our ultimate goal, however, was to identify curricular adaptations needed to ensure relevance, feasibility, and appropriateness of educational content to the target setting in which pediatric resuscitation education is to be conducted. This process enabled us to implement fully contextualized curricula in two very different international venues.

## Materials and Methods

### Tool Development

The original PIPES tool has 105 data items divided into five sections and functions as a scoring tool to compare the surgical capacity of different health-care facilities by calculating the PIPES Index (26, 38). The index can be calculated by tallying the total scores obtained in the survey. The Personnel section queries the total number of key practitioners (e.g., surgeons). The infrastructure, procedures, equipment and supplies sections have a binary response system of either “Always Available” versus “Not Always Available” or “Done” versus “Not Done.”

We modified the PIPES tool in order to assess the pediatric medical resuscitation capacity of health-care facilities in resource-limited settings. As our tool was to be used for contextualization of a curriculum as opposed to rating facility capacity, we omitted the scoring component of the tool and replaced it with qualitative open-ended questions in order to garner more details about the hospital environments. We omitted items that were specific to surgical capacity and added items relevant to pediatric medical resuscitation. We also replaced the binary response system with a three-option response system (i.e., always available, usually available or seldom/never available) in order to account for potential variability in the availability of resources at different sites. Finally, we omitted the “Personnel” section, as this was specific to measuring overall hospital operational capacity and was less relevant for the curriculum development process.

In order to ensure that our curriculum addressed clinical topics of importance to our learners, we added a Section “Epidemiology.” In this section, we queried the most common causes of potentially preventable death among children in the local practice setting. We also queried survey respondents to elucidate their perceived learning needs and prior pediatric resuscitation training and experience. We also added a Section “Current Practices/Skills,” which used a five-point Likert scale to measure the confidence of survey respondents in managing emergency clinical scenarios and performing emergency procedures (see Appendix S1 in Supplementary Material for final iteration of tool used in the study—the PIPES Tool for Assessment of Pediatric Resuscitation Capacity in LMIC).

### Tool Implementation

#### Study Settings

We implemented our needs assessment tool at two locations: Yangon Children’s Hospital (YCH) in Yangon, Myanmar and Makerere University College of Health Sciences (MUCHS) in Kampala, Uganda. The study proposal was reviewed by the Institutional Review Board (IRB) of Johns Hopkins University School of Medicine and was considered to be exempt. In addition, local site approval to conduct the study was obtained from the ethics board and/or training committee leadership at both YCH and MUCHS. The two study sites were selected for convenience reasons, due to existing institutional partnerships.

#### Study Participants

At YCH in Myanmar, intended study participants consisted of first, second, and third year postgraduate trainees in Pediatrics, as well as a group of general practitioners pursuing a 1-year Diploma in Child Health course. At MUCHS, intended study participants consisted of second and third year postgraduate trainees in Pediatrics.

#### Data Collection

The needs assessment survey was administered to all participants immediately prior to the start of a resuscitation course. The paper-based survey was administered in person at both sites.

#### Data Analysis and Use of Results

Results were analyzed using simple descriptive statistics in Microsoft Excel. Results were used to make modifications to the planned curriculum as needed in order to appropriately contextualize the content in each setting. Of note, the investigators completed multiple phone meetings with local stakeholders as well as in-person visits to both sites prior to administration of the survey. This process allowed development of the majority of the curriculum in advance of the needs assessment survey. Survey results were then used in an iterative fashion to “fine-tune” and modify the curriculum at subsequent sites in order to optimize feasibility of recommendations and relevance to the local clinical context.

## Results

A total of 94 participants completed the pre-course needs assessment survey, 62 in Myanmar and 32 in Uganda (100% response rate). Age range of respondents was 25–40 years. 74% of respondents were in their second or third postgraduate year of pediatric specialty training. Further results are presented below by category.

### Infrastructure

**Figure 1:**
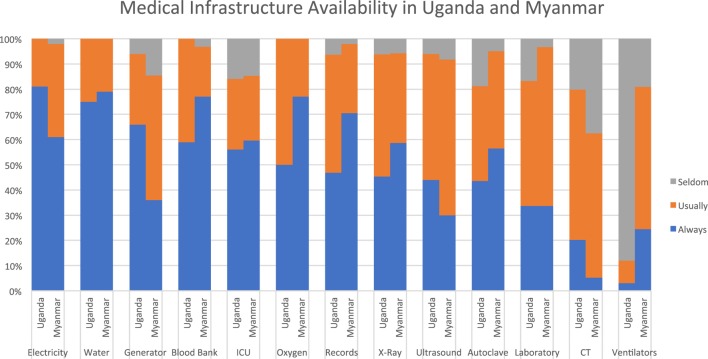
Infrastructure availability.

At both sites, most infrastructure items were described as being “always” or “usually” available by the majority of respondents. The one exception was ventilators at the Uganda site, which were described as “seldom/never” available by 88%. Compared to Uganda, respondents in Myanmar reported more variability in availability of electricity. The vast majority at both sites reported they “always” or “usually” had access to specialized services including blood bank, intensive care, and medical records. While no respondents described oxygen as “seldom/never” available, it is notable that only 50% in Uganda and 77% in Myanmar described it as “always” available. Access to diagnostic testing was limited at both sites. X-ray was the most commonly available imaging modality, cited as “always available” by 44% in Uganda and 61% in Myanmar. Laboratory services and advanced imaging were described as “usually” or “seldom” available by a majority at both sites. Ventilators were much more available in Myanmar compared to Uganda (see Figure 1).

### Procedures

**Figure 2:**
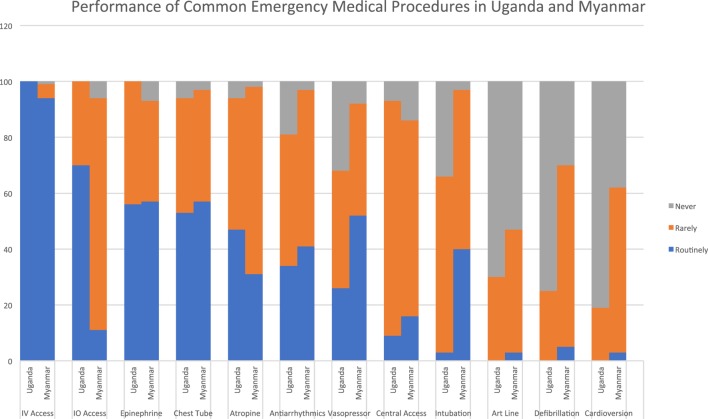
Common emergency procedures performed.

Peripheral intravenous access was the only procedure described as being performed “routinely” at both sites. In Uganda, 70% of respondents reported that intraosseous access was performed “routinely,” compared to Myanmar where 83% indicated that this procedure was performed “rarely.” Over half of respondents at both sites described administering epinephrine “routinely.” In Myanmar, a similar number reported “routinely” administering vasopressor infusions, while this proportion was much smaller in Uganda. At both sites, a sizeable minority of respondents described use of atropine and antiarrhythmics as routine. Chest tube placement was the most commonly performed invasive procedure, with a majority at both sites describing it as routine. Intubation was much more common in Myanmar than in Uganda, with 40% of respondents indicating that they perform this procedure “routinely” in Myanmar compared to only 3% in Uganda. Central venous access, arterial line placement and electrical therapies for the heart were rare in both settings (see Figure 2).

### Equipment

**Figure 3:**
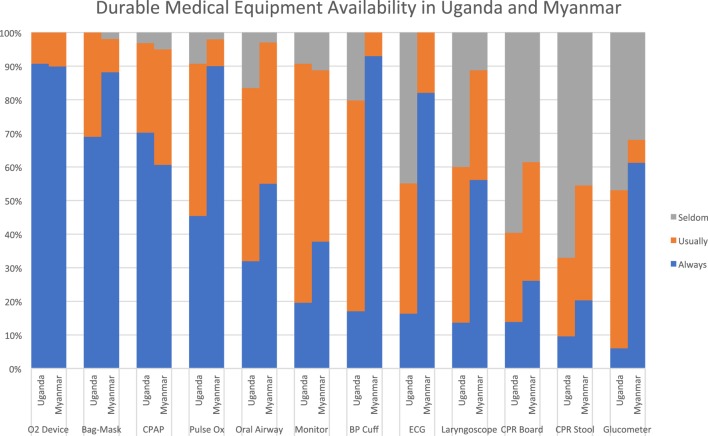
Equipment availability.

Most respondents at both sites reported that noninvasive respiratory support equipment was “always” or “usually” available, including oxygen delivery devices, bag-valve-mask ventilation devices, and continuous positive airway pressure systems. Diagnostic equipment like pulse oximeters, blood pressure cuffs, electrocardiogram machines, and glucometers were much more readily available in Myanmar than Uganda, where access to these items was extremely limited. Respondents from both sites reported limited access to cardiac monitors, with only 19% in Uganda and 37% in Myanmar reporting them as “always available.” Respondents in Myanmar reported better availability of advanced airway adjuncts compared to those in Uganda, although access to these items was suboptimal in both settings. Both sites reported very limited availability of quality CPR equipment like backboards and stepstools (see Figure 3).

### Supplies

**Figure 4:**
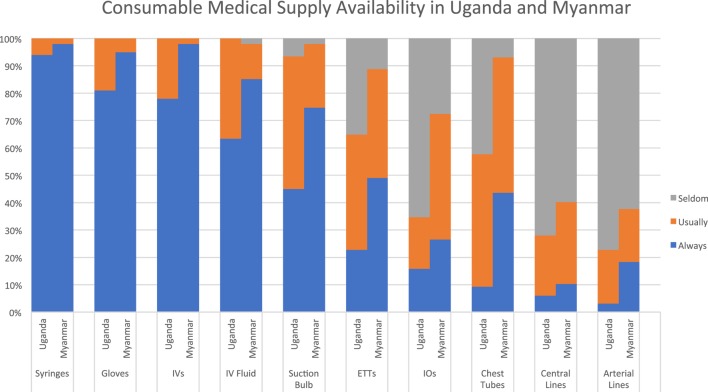
Consumable supplies availability.

All respondents at both sites indicated that basic supplies like syringes, gloves, and intravenous catheters were “always” or “usually” available. While this was also true for intravenous fluids, a significant proportion at each site (34% in Uganda and 15% in Myanmar) indicated that this essential supply is not always available. Similarly, infant suction bulbs were not uniformly available at either site. Both sites reported a lack of access to intraosseous needles, with 66% in Uganda and 27% in Myanmar reporting that these are “seldom/never” available. Higher-level procedural equipment was relatively unavailable in both settings, with endotracheal tubes more readily available than invasive vascular access devices and chest tubes (see Figure 4).

### Epidemiology

**Figure 5:**
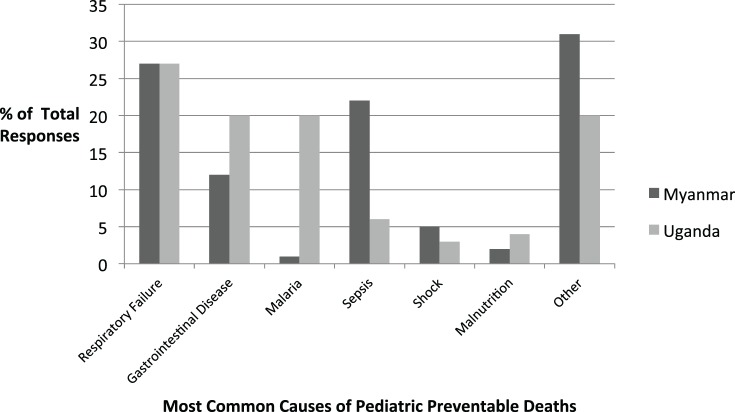
Most commonly cited causes of pediatric preventable death in survey participants’ clinical settings.

Infectious diseases were listed as the predominant cause of preventable death in children at both the Myanmar and Uganda sites (see Figure 5). Specifically, respiratory infections occupied the top position at both sites, and gastrointestinal diseases were also common. Differences in local epidemiology were seen between the two sites, as malaria was frequently cited as a major cause of preventable death in Uganda but was rarely noted in Myanmar. Sepsis, on the other hand, was frequently cited in Myanmar but not in Uganda.

### Self-Identified Learning Needs

At the Myanmar site, the three most commonly cited learning needs were in the categories of airway/breathing management (28% of respondents), circulation/fluid resuscitation (22%), and cardiac arrest management (16%). Within each of these categories, the most frequently cited learning needs were intubation (54%), intraosseous needle (IO) placement (65%), and defibrillation/cardioversion (38%). At the Uganda site, the three most commonly cited learning needs were in the categories of general resuscitation principles (27%), cardiac arrest management (22%), and circulation/fluid resuscitation (19%). Within each of these categories, the most frequently cited learning needs were basic neonatal/pediatric resuscitation topics (65%), defibrillation/cardioversion (33%), and central venous cannulation (17%).

### Pediatric Resuscitation Experience

At the Uganda site, 87.5% of participants stated that they had prior formal training in pediatric resuscitation, 75% of whom stated that the course involved either simulation or hands-on skills practice. At the Myanmar site, only 63% of participants stated that they had prior training, only 50% of whom reported simulation or hands-on skills practice as part of this training. 97% of participants in Uganda indicated that they managed critically ill children either “daily” or “regularly,” compared to only 48% in Myanmar (see Table 1).

**Table 1:** Frequency of exposure to critically ill children in clinical settings.

	Myanmar, % respondents	Uganda, % respondents
Daily (4 or more times per week)	8	75
Regularly (1–2 times per week)	40	22
Occasionally (1–2 times per month)	40	0
Rarely (1–2 times per year)	3	0
No response	9	3

### Current Practices/Skills

The clinical scenarios that participants reported the greatest confidence in managing were similar at both sites: respiratory distress and shock. The clinical scenarios that the participants were “least confident” managing were also similar: cardiac arrest and pneumothorax. See Table 2 for detailed results. Participants in Uganda reported higher confidence overall compared to those in Myanmar.

**Table 2:** Clinical scenario management confidence scores.

	Uganda, mean Likert Score (SD)	Myanmar, mean Likert Score (SD)
Bradycardia	3.7 (0.9)	3.4 (1.2)
Cardiac arrest	3.2 (1.0)	2.3 (1.2)
Decreased level of consciousness	4.2 (0.8)	3.4 (1.0)
Hypoxia	4.0 (0.7)	3.6 (0.9)
Respiratory distress	4.3 (0.7)	3.6 (0.9)
Seizures	4.4 (0.6)	3.7 (1.0)
Shock	4.3 (0.6)	3.8 (0.9)
Tachycardia	3.6 (1.0)	3.4 (1.2)

Likert scale consisted of numeric options ranging from 1 to 5:1 = “Not at all confident” 3 = “Somewhat confident” and 5 = “Very confident.”

The procedures that the participants reported highest confidence in performing were the same at both sites, with differences only in rank order: airway assessment, breathing assessment, circulation assessment, and IV placement. The procedures that the participants were least confident performing also had some similarities between the sites. Participants at both sites were not confident performing central line placement, defibrillation/cardioversion, and intubation. See Table 3 for detailed results.

**Table 3:** Procedural confidence scores.

	Uganda, mean (SD)	Myanmar, mean (SD)
Airway assessment	4.2 (0.7)	4.0 (0.9)
Breathing assessment	4.3 (0.7)	4.0 (0.9)
BVM ventilation	4.0 (0.8)	3.8 (0.9)
Central line placement	1.9 (1.3)	1.3 (0.8)
Circulation assessment	4.3 (0.8)	4.0 (0.9)
CPR	3.9 (0.7)	3.7 (0.9)
Defibrillation/cardioversion	1.4 (0.7)	1.4 (0.8)
EKG or cardiac rhythm interpretation	2.1 (1.1)	2.9 (0.8)
Intraosseous line placement	4.2 (1.0)	2.4 (1.3)
Intubation	2.1 (1.1)	2.1 (1.0)
IV placement	4.6 (0.6)	3.9 (1.0)

Likert scale consisted of numeric options ranging from 1 to 5:1 = “Not at all confident” 3 = “Somewhat confident” and 5 = “Very confident.”

## Discussion

Our modified PIPES tool was administered to a group of physician learners in Myanmar and Uganda as part of a needs assessment process for the design and implementation of a contextualized curriculum in pediatric resuscitation using simulation-based pedagogy. We were able to achieve a 100% response rate to the needs assessment survey, which was achievable due to in-person administration of the survey immediately prior to the start of the course. As the purpose of collecting these data was to inform the curriculum development process, we reviewed the results with the goal of optimally contextualizing our training program to each setting. While it would be impossible to exhaustively describe every curricular adaptation made in each setting, Table 4 offers several key examples of how these data were used to optimize applicability of our educational content for the target learner groups.

**Table 4:** Examples of key findings and their use in curriculum development.

Finding	Action
Absence of ventilators and laryngoscopes in Uganda	Focused on effective noninvasive ventilation and omitted advanced airway skills from curriculum at this site

Limited access to cardiac monitors at both sites	Emphasized frequent clinical reassessment to determine response to treatment and trajectory of condition

Variable access to diagnostic support tools, including pulse oximetry, blood pressure cuffs, glucometers, and laboratory services (particularly in Uganda)	Emphasized established clinical criteria for empiric administration of oxygen, IV fluid, glucose, and blood products (major focus of training in Uganda)

Rare performance of cardioversion or defibrillation at either site (related to lack of defibrillators)	Despite participants’ self-identified learning needs, chose to omit these skills from curriculum at both sites

Lack of availability of intraosseous needles at both sites	Addressed improvization of equipment for intraosseous access when needed

Relatively lower confidence in intraosseous needle placement in Myanmar	Spent additional time on this skill at this site

Relatively lower confidence in CPR performance at both sites	Allotted significant time to this skill at both sites, and incorporated CPR quality feedback tools to optimize learner performance

Frequent mortality associated with malaria in Uganda	Added entire module about life-threatening complications of malaria for this site

Frequent use of epinephrine at both sites	Emphasized alternate appropriate treatments, particularly respiratory support for bradycardia, and volume expansion for shock

In applying our results from the needs assessment to refine the curriculum, we took into account a prior systematic review of resuscitation training in developing countries, which demonstrated that important educational outcomes are inconsistently defined and that training courses are often modified to available training resources, rather than to the needs and resources of the local health-care setting and patients (40). Training that is not tailored to local needs perpetuates a “common misconception that emergency medical care is inherently expensive and requires high-technology interventions as opposed to simple and effective strategies” (41). This needs assessment was an effort to facilitate essential contextualization of the curriculum in both settings. The process by which the needs assessment was used to tailor simulation scenarios and skills stations to the local settings and resource availability was analogous to the process used in similar studies in Africa that utilized a shorter assessment tool known as the facilities readiness assessment (18). The modified tool used in our study allowed for a more extensive evaluation of the local health-care resources specifically applicable to pediatric resuscitation and proved effective in understanding the local landscape of our target learners in both Uganda and Myanmar. Below, we highlight notable findings from the results and how they compare with what is already known in the literature.

### Epidemiology—Local Disease Burden

**Figure 6:**
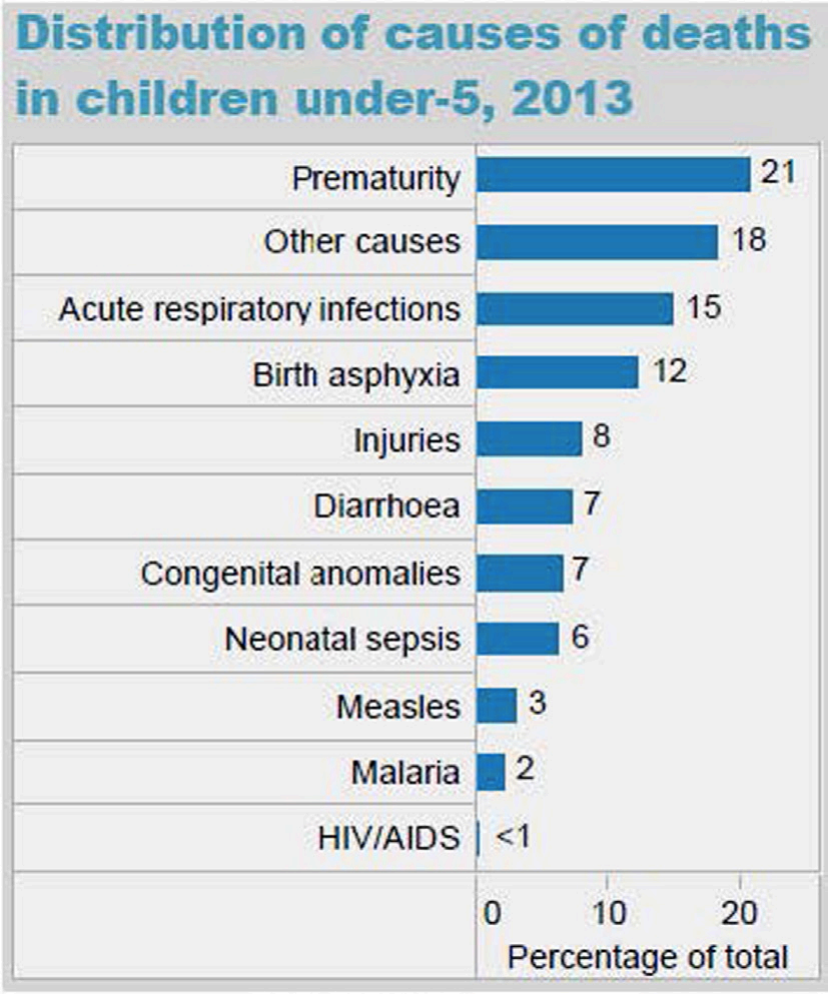
World Health Organization (WHO) distribution of causes of death in children under five in Myanmar (Source: country statistics and global health estimates by WHO and UN partners http://www.who.int/gho/countries/mmr.pdf).

**Figure 7:**
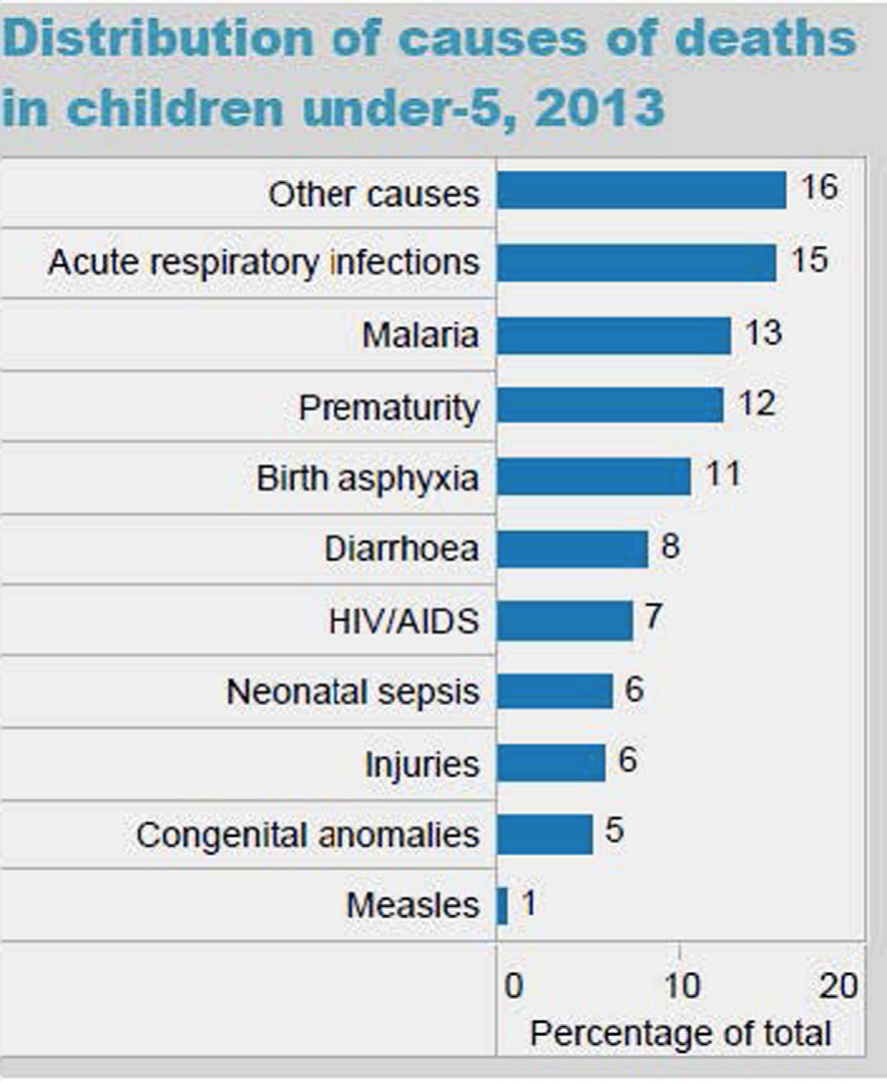
World Health Organization (WHO) distribution of causes of death in children under five in Uganda (Source: country statistics and global health estimates by WHO and UN partners http://www.who.int/gho/countries/uga.pdf).

The epidemiology section of the survey allowed us to determine if the national morbidity and mortality statistics provided by the WHO were reflected in the local pediatric population served by our target learners. In Myanmar, the majority of the causes of under five mortality rates (UFMR) listed in WHO national statistics were mentioned by survey respondents, although the ranking of diseases differed somewhat. One of the major differences was that in the 2013 WHO statistics, neonatal sepsis was listed as the seventh leading cause of death in Myanmar, whereas in our survey, it was the second most common response for preventable causes of mortality (see Figure 6). In Uganda, many of the causes of death listed in the WHO national statistics were also mentioned by the survey respondents, with rankings also differing somewhat (see Figure 7). Some important causes of death listed by WHO were not mentioned by survey respondents, including prematurity, HIV/AIDS, and injuries. Of note, our survey asked for causes of *preventable* death. Our Ugandan learners, working in a severely resource-constrained environment, may encounter these causes of death routinely, but simply not perceive them as preventable. Our curriculum ultimately incorporated both locally identified and WHO-cited causes of pediatric mortality. Our educational team felt strongly that it was important to design simulation scenarios targeting causes of UFMR identified through an emic or local perspective in order to enhance face validity of the curriculum with target learners.

### Pediatric Resuscitation Training and Prior Clinical Exposure

The majority of participants at both local sites had some form of prior pediatric resuscitation training. Interestingly, this is in contrast with many other studies that have reported on baseline knowledge prior to training courses (40), perhaps because our survey was conducted at facilities that had formal residency training programs in pediatrics. Prior exposure to hands-on skills practice and/or simulation was more variable and differed between the two sites. In Myanmar, only one-third of participants had prior simulation-based resuscitation training, despite the fact that they resuscitate children on a fairly regular basis.

The percentage of survey participants who indicated that they managed critically ill children on a daily basis was significantly higher in Uganda compared to Myanmar, and this was reflected in the higher confidence scores reported by Ugandan participants. Participants at both sites reported frequent exposure to critically ill children, reinforcing the importance of formal training and frequent refreshers in pediatric and neonatal resuscitation skills for both cohorts of clinicians.

### Self-Identified Learning Needs

Airway management was listed as a learning need for a significant percentage of participants in both the Uganda and Myanmar cohorts. This is not surprising given that respiratory illnesses and respiratory failure were the most frequently listed causes of preventable death at both sites. The self-identified learning needs of airway and cardiac arrest management were reflected in the low mean confidence scores for these scenarios at both sites, as well as for related skills such as intubation and defibrillation/cardioversion. However, it was noted in the PIPES survey that the necessary equipment and supplies for these procedures were often not available, raising the question of whether these skills are truly contextually relevant, but also likely leading to minimal practical use of these skills and, hence, lower confidence scores.

Although this study was conducted in resource-limited settings, the major learning needs we identified were consistent with self-identified knowledge gaps in a recent large scale survey of over 700 emergency medical care providers for children in rural settings in the United States, where resource limitations are often also present (42). These gaps included pediatric airway management and procedural skills in neonatal resuscitation and intraosseous access. This concordance with the literature even in a developed setting highlights the importance of addressing specific educational needs for health-care providers to care for neonatal and pediatric populations.

### Impact on Curriculum Design

The sections of the needs assessment survey that had the greatest impact on the curriculum instructional design were the local epidemiology, followed by the available equipment and infrastructure in each setting. These data allowed us to focus the curriculum in each location on high-yield material that was relevant to the learners and the population they serve. For example, in Uganda, one learning station focused on management of malaria according to local guidelines. In Myanmar, this was not needed, but a learning station did focus on resuscitation in dengue shock, a common cause of morbidity and mortality in the Southeast Asian region (see Table 4 for examples).

Participants’ self-identified learning needs were also taken into account, but coverage of these topics emphasized techniques and procedures that could be performed with available equipment based on the PIPES survey. For example, due to the lack of defibrillators, central venous catheters, arterial line catheters, and the inconsistent availability of ventilators at both sites, the procedural skills of defibrillation/cardioversion, central line placement, arterial line placement, and intubation/advanced airway management were de-emphasized. Greater emphasis was placed on clinical assessment skills, effective noninvasive airway management (e.g., high quality BVM ventilation), and management of shock using available local equipment.

In order to ensure relevance and feasibility, we taught procedures using locally available equipment. For example, emergency intraosseous (IO) access was taught using bone marrow needles or other large-bore devices rather than with the commercially available IO needles used in the United States. When certain equipment was not available in the local setting, the educational team worked with the local team to design workable alternatives. When equipment for certain procedures is not available, it is also essential for trainers to understand if and how clinicians improvize in order to perform these procedures. For example, our respondents reported very limited access to chest tubes, yet a majority indicated that chest tube placement is a “routinely” performed procedure. Had chest tube placement been an objective of our curriculum, it would have been essential to explore how clinicians perform this procedure in the absence of purpose-specific equipment and to incorporate locally available supplies and techniques in the teaching of this skill.

When designing simulation scenarios, we took care to mimic resource constraints in the actual clinical environment and to require learners to address these issues in their management. For example, glucometers were scarce in Uganda, while they were more readily available in Myanmar. In our hypoglycemia scenarios, we provided a finger-stick glucose reading for participants in Myanmar, but required participants in Uganda to rely on clinical assessment to determine the need for glucose administration. Similarly, the 10% dextrose (D10) solutions used for hypoglycemia in young children are not commercially available in Uganda, necessitating that clinicians mix their own solutions using sterile water and higher concentration dextrose. In order to ensure mastery of this skill, our hypoglycemia simulation included mixing of D10 from locally available products as a critical action. Subtle alterations such as these ensured contextual relevance of our scenarios and reinforced essential skills required of health-care providers in their actual clinical settings.

Overall, the data acquired through our survey allowed for the curriculum to be tailored to local needs and resources to increase its feasibility, applicability, and impact. This practice is consistent with recommendations in the literature to appropriately calibrate and contextualize resuscitation courses from developed countries in order to ensure feasibility in resource-limited settings, thereby strengthening the chain of survival from within the local health-care system (17, 20, 41).

### Limitations and Next Steps

The needs assessment survey in this study was implemented at only two sites in East Africa and Southeast Asia, and therefore its generalizability may be limited to sites with similar learning needs and epidemiology. Furthermore, our participants were subspecialty trainees based at referral centers, so our tool may be less applicable for first-line providers and facilities. Although the needs assessment is based on a WHO tool that has been validated in multiple resource-limited settings, our modified tool has not yet undergone validity testing. This will be a goal of future studies. In addition to tool validation, our next steps will include the further revision of existing simulation-based curricula in pediatric resuscitation to be more appropriately contextualized to local epidemiology, learning needs, and available resources.

## Conclusion

The WHO Tool for Situational Analysis to Assess Emergency and Essential Surgical Care and the PIPES tools were successfully adapted to assess pediatric resuscitation capacity and learning needs in two different clinical settings. The implementation of the tool in two different countries in Asia and Africa helps to improve its generalizability for LMIC settings in the developing world. Results from the needs assessment at each site provided the basis for design of a pragmatic and appropriately contextualized curriculum in neonatal and pediatric resuscitation at each site, as well as a process for iterative revision of the curriculum for subsequent courses. These results informed curriculum development and instructional design, enabling the team to address self-identified learning needs, while emphasizing high-yield procedural skills that were of practical value, given the local resource limitations. The results also improved engagement of local stakeholders and participants in the curriculum, as they perceived that they had input into successive iterations of the curriculum through their responses to the needs assessment survey. The approach of partnering with local educational stakeholders in the curriculum design process through a formal needs assessment is consistent with recommendations by the Lancet Commission in 2010 in their publication *Health professionals for a new century: transforming education to strengthen health systems in an interdependent world* (43). These recommendations include reformation of local competencies through global flows of knowledge and cultivation of ownership through local clinician leadership in shaping educational processes. This concept provided a foundation for the ultimate design of our curriculum based on our needs assessment survey. Future studies will focus on validation of the PIPES tool in its modified form and dissemination of the tool to other resource-limited settings, in addition to implementation and evaluation of subsequent curricular interventions.

## Ethics Statement

This study was carried out in accordance with the recommendations of the Johns Hopkins University School of Medicine Institutional Review Board (JHUSOM IRB). The study protocol was determined to be exempt research by the JHUSOM IRB under the DHHS regulations (i.e., research conducted in established or commonly accepted educational settings, involving normal educational practices, such as (i) research on regular and special education instructional strategies or (ii) research on the effectiveness of or the comparison among instructional techniques, curricula, or classroom management methods). In addition, local site approval and consent for conduct of the study in Uganda and Myanmar were obtained from the Head of the Department of Paediatrics and the Director of Training and Education at Children’s Hospital of Yangon, Myanmar, as well as from the ethics board of Mulago Hospital and Makerere University College of Health Science in Kampala, Uganda.

## Author Contributions

NS, AC, JR, and JJ conceived of the needs assessment survey, implemented the survey in each setting, performed data analysis, and contributed to the writing of the manuscript. TM, YK, and SG assisted in implementation of the survey at each site and assisted in data collection. LC assisted in data analysis and writing of the manuscript. All authors approved the final version of the manuscript.

## Conflict of Interest Statement

The authors declare that the submitted work was carried out without any personal, professional or financial relationships that could be construed as a conflict of interest.
