# Supplementary material for: A Standardized Needs Assessment Tool to Inform the Curriculum Development Process for Pediatric Resuscitation Simulation-Based Education in Resource-Limited Settings
Source: Front Pediatr. 2018 Feb 28;6:37. doi: 10.3389/fped.2018.00037 (PMC5863499; doi:10.3389/fped.2018.00037)
Supplement: Supplementary file 1 [file data_sheet_1.docx]

**Appendix 1: PIPES Tool for Assessment of Pediatric Resuscitation Capacity in LMIC**

**Please tell us about yourself:**

| Student Number |  |
| --- | --- |
| Age |  |
| Number of years in postgraduate training (count from graduation from medical school) |  |

**EPIDEMIOLOGY**

1. Please list the three most common causes of death among pediatric patients in your practice

a.____________________________________________________________________________

b.____________________________________________________________________________

c.____________________________________________________________________________

1. Please list three concepts or skills you would like to learn more about in order to be more effective in managing critically ill children in your practice:

a.____________________________________________________________________________

b.____________________________________________________________________________

c.____________________________________________________________________________

1. Have you taken a pediatric resuscitation or life support course before? YES NO

If yes, please describe: ___________________________________________________________

1. If yes, did that course include simulation or hands-on skills practice? YES NO
2. Have you taught pediatric resuscitation or life support skills to others? YES NO
3. Would you be interested in teaching these skills to others in the future? YES NO

(medical students, junior trainees, community providers, etc.)

1. Would you be willing for us to contact you in the future? YES NO
2. How often do you see critically ill children in your practice? RARELY (1-2 times/year)

OCCASIONAL (1-2 times/month) REGULARLY (1-2 times/week) DAILY (4 or more times per week)

**PIPES (Personnel, Infrastructure, Procedures, Equipment, Supplies)**

9. Please indicate whether each of the resources listed below is available in your practice setting:

| **INFRASTRUCTURE** | Always Available | Usually Available | Seldom/Never Available |
| --- | --- | --- | --- |
| Running water |  |  |  |
| Oxygen |  |  |  |
| Electricity |  |  |  |
| Back-up generator |  |  |  |
| Medical records |  |  |  |
| Lab to test blood and urine |  |  |  |
| Blood bank |  |  |  |
| X-ray machine |  |  |  |
| Ultrasound machine |  |  |  |
| CT scanner |  |  |  |
| Ventilators |  |  |  |
| Pediatric/neonatal intensive care unit |  |  |  |
| Autoclave/sterilizer |  |  |  |

10. Please indicate how often each of these procedures are performed at your hospital:

| **PROCEDURE** | Routinely | Rarely | Never |
| --- | --- | --- | --- |
| Oropharyngeal airway placement |  |  |  |
| Endotracheal intubation |  |  |  |
| Peripheral venous access |  |  |  |
| Central or umbilical venous access |  |  |  |
| Intraosseous access |  |  |  |
| Arterial line placement (invasive blood pressure monitoring) |  |  |  |
| Needle decompression of pneumothorax |  |  |  |
| Chest tube placement |  |  |  |
| Foley catheter placement |  |  |  |
| Pericardiocentesis |  |  |  |
| Bedside ultrasound |  |  |  |
| Defibrillation |  |  |  |
| Synchronized cardioversion |  |  |  |
| External cardiac pacing |  |  |  |
| Administration of epinephrine |  |  |  |
| Administration of atropine |  |  |  |
| Administration of antiarrhythmic drugs |  |  |  |
| Administration of vasopressor infusions |  |  |  |

11. Please indicate whether each piece of durable equipment listed below is available in your practice setting:

| **EQUIPMENT** | Always Available | Usually Available | Seldom/Never  Available |
| --- | --- | --- | --- |
| Bag valve and mask (pediatric sizes) |  |  |  |
| Oral or nasopharyngeal airways (pediatric sizes) |  |  |  |
| Laryngoscope (pediatric sized blades) |  |  |  |
| Pulse oximeter |  |  |  |
| Noninvasive ventilation (CPAP/BiPAP) |  |  |  |
| Oxygen cannula/mask |  |  |  |
| Backboard (for CPR) |  |  |  |
| Step stool (for CPR) |  |  |  |
| Cardiac monitor |  |  |  |
| Blood pressure measurement (pediatric sized cuffs) |  |  |  |
| Weight scale (infant) |  |  |  |
| Broselow/weight based Tape |  |  |  |
| Glucometer |  |  |  |
| Electrocardiogram Machine |  |  |  |
| Incubators |  |  |  |
| Defibrillator |  |  |  |

12. Please indicate whether each disposable supply listed below is available in your practice setting:

| **SUPPLIES** | Always Available | Usually Available | Seldom/Never  Available |
| --- | --- | --- | --- |
| Gloves (examination) |  |  |  |
| Gloves (sterile) |  |  |  |
| IV access supplies |  |  |  |
| Syringes |  |  |  |
| Endotracheal tubes (pediatric sizes) |  |  |  |
| Suction bulb (for infants) |  |  |  |
| Central Line kits/supplies |  |  |  |
| Arterial Line kits/supplies |  |  |  |
| Intra-osseous needles |  |  |  |
| Sterile IV fluid bags |  |  |  |
| Chest tubes |  |  |  |

**CURRENT PRACTICES/ SKILLS**

***Clinical Scenario Confidence***

13. Please rate your confidence in assessing and managing each clinical scenario listed below:

| How confident are you treating a child with… | 1  Not At All Confident | 2 | 3  Somewhat  Confident | 4 | 5  Extremely Confident |
| --- | --- | --- | --- | --- | --- |
| Cyanosis |  |  |  |  |  |
| Hypoxia |  |  |  |  |  |
| Bradycardia (decreased heart rate) |  |  |  |  |  |
| Tachycardia (increased heart rate) |  |  |  |  |  |
| Asystole |  |  |  |  |  |
| Ventricular tachycardia |  |  |  |  |  |
| No recordable blood pressure |  |  |  |  |  |
| Seizures |  |  |  |  |  |
| Respiratory distress |  |  |  |  |  |
| Pneumothorax |  |  |  |  |  |
| Decreased level of consciousness |  |  |  |  |  |
| Hypotension or shock |  |  |  |  |  |

***Procedural Confidence***

14. Please rate your confidence in performing each of the procedures listed below on children:

| How confident are you performing… | 1  Not At All Confident | 2 | 3  Somewhat  Confident | 4 | 5  Extremely Confident |
| --- | --- | --- | --- | --- | --- |
| Airway assessment |  |  |  |  |  |
| Breathing assessment |  |  |  |  |  |
| Circulation assessment |  |  |  |  |  |
| Cardiopulmonary resuscitation |  |  |  |  |  |
| Bag-valve-mask ventilation |  |  |  |  |  |
| Defibrillation and Cardioversion |  |  |  |  |  |
| IV placement |  |  |  |  |  |
| Intraosseous line placement |  |  |  |  |  |
| Central line placement |  |  |  |  |  |
| Electrocardiogram or cardiac rhythm interpretation |  |  |  |  |  |
| Intubation |  |  |  |  |  |
| Needle decompression of pneumothorax |  |  |  |  |  |
| Placement of a chest tube (or thoracostomy tube) |  |  |  |  |  |
